# Investigating the Prognostic Role of Peripheral Inflammatory Markers in Mild Cognitive Impairment

**DOI:** 10.3390/jcm12134298

**Published:** 2023-06-27

**Authors:** Giacomo Tondo, Davide Aprile, Fabiola De Marchi, Barbara Sarasso, Paola Serra, Giordana Borasio, Esther Rojo, Juan Francisco Arenillas, Cristoforo Comi

**Affiliations:** 1 Neurology Unit, Department of Translational Medicine, Sant’Andrea Hospital, University of Piemonte Orientale, Corso Abbiate 21, 13100 Vercelli, Italy; aprile.davide@hotmail.com (D.A.); cristoforo.comi@med.uniupo.it (C.C.); 2 Centre for Dementia and Cognitive Disorders, Sant’Andrea Hospital, Corso Abbiate 21, 13100 Vercelli, Italy; barbara.sarasso@aslvc.piemonte.it (B.S.); paola.serra@aslvc.piemonte.it (P.S.); giordana.borasio@aslvc.piemonte.it (G.B.); 3 Neurology Unit, Department of Translational Medicine, Maggiore della Carità Hospital, University of Piemonte Orientale, 28100 Novara, Italy; fabiola.demarchi@uniupo.it; 4 Department of Neurology and Medicine, Hospital Clínico Universitario, Universidad de Valladolid, 47003 Valladolid, Spain; erojo80@yahoo.it (E.R.); juanfrancisco.arenillas@uva.es (J.F.A.); 5 Interdisciplinary Research Center of Autoimmune Diseases (IRCAD), University of Piemonte Orientale, 28100 Novara, Italy

**Keywords:** neutrophil-lymphocyte ratio, cognitive impairment, dementia, neuroinflammation

## Abstract

Growing evidence suggests that neuroinflammation plays a critical role in the pathogenesis of neurodegenerative diseases. Peripheral markers of inflammation, including blood cell counts and their ratios, such as the neutrophil-to-lymphocyte ratio (NLR), have been reported as an easily accessible and reliable proxy of central nervous system inflammation. However, the role of peripheral inflammation in dementia and Mild Cognitive Impairment (MCI) still needs to be clarified. In the current study, we aimed to assess the prognostic role of the NLR and other peripheral markers of inflammation in a sample of 130 amnestic MCI, followed up for two to five years. The Mini-Mental state examination (MMSE) score at baseline and follow-up visits was used to assess global cognitive status at each visit and the degree of cognitive decline over time. Baseline peripheral markers of inflammation included blood cell counts and ratios, specifically the NLR, the platelet-to-lymphocyte ratio (PLR), the monocyte-to-lymphocyte ratio (MLR), and the systemic immune inflammation index (SII). After classifying subjects into CONVERTERS and non-CONVERTERS (respectively, patients converting to dementia and subjects showing stability at the last available follow-up), we compared peripheral markers of inflammation among groups ed correlated them with cognitive measures, testing the ability of significant factors to predict conversion to dementia. In our cohort, CONVERTERS showed lower baseline MMSE scores (*p*-value = 0.004) than non-CONVERTERS. In addition, CONVERTERS had statistically elevated NLR (*p*-value = 0.005), PLR (*p*-value = 0.002), and SII levels (*p*-value = 0.015), besides a lower number of lymphocytes (*p*-value = 0.004) compared with non-CONVERTERS. In a logistic regression analysis, baseline MMSE scores and NLR predicted conversion to dementia. Tertiles analysis showed that MCI with the highest NLR values had a higher conversion risk. Our study supports the hypothesis that a dysregulation of peripheral inflammation involving both lymphocytes and neutrophils may play a role in the pathogenesis of dementia, even at the early stages of neurodegeneration, as in the MCI condition.

## 1. Introduction

Dementia is a progressive neurological disorder provoking a devasting impairment of cognitive functions and behaviors, affecting the ability to carry out everyday activities. Dementia is included among the five most burdensome conditions among the elderly; currently, more than 50 million people live with dementia worldwide, and this impressive number is estimated to almost triplicate by 2050 [1]. Due to the social and economic impact of dementia, the World Health Organization recognized dementia as a public health priority [2]. Alzheimer’s disease (AD) represents the most frequent etiology of dementia and is among the ten most common causes of death globally [3]. Advanced age is considered the most significant risk factor for AD; thus, the prevalence of AD and dementia is continuously increasing due to the progressive aging of the world’s population, representing a healthcare burden of epidemic proportions [2].

Mild Cognitive Impairment (MCI) is an intermediate condition between physiological aging and dementia [4]. According to the clinical presentation, MCI subjects can be classified into two groups: amnestic MCI, showing impairment in memory, and non-amnestic MCI, manifesting impairment in cognitive domains other than memory, including language, executive functions, or visuospatial abilities. The amnestic MCI is usually considered the prodromal phase of AD due to the high likelihood of converting to AD dementia [5]. MCI prevalence is variable among studies due to the use of different diagnostic criteria and heterogenous populations [6], but it is reported to increase with age, reaching about 15% for people aged 75–79 years and 25% for people aged 80–84 years [7]. The estimated cumulative incidence of the development of dementia in MCI subjects over 65 years is 14.9% after two years [7]. However, not every subject with MCI is doomed to convert to dementia, and some may remain stable over time or revert to normal cognition [8,9]. Thus, identifying markers able to predict the stability or conversion is crucial when considering MCI; in addition, due to the large prevalence of this condition, it is critical to consider the accessibility of the considered markers and the possibility of testing large populations with low costs.

Despite remarkable progress in understanding the pathogenic mechanisms underlying the neurodegenerative processes leading to dementia, a clear picture is still far from being achieved. Several environmental factors, lifestyle, and genetics may be involved. Increasing evidence supports a critical role for dysregulation in neuroinflammatory responses, favoring abnormal protein deposition, excessive oxidative stress, and mitochondrial dysfunction [10]. Historically, the brain has been considered an immune-privileged organ, mainly due to the presence of the blood-brain barrier (BBB); recently, this dogma has been revised by the evidence that pathological inflammation in the brain, linked to the breakdown of the BBB, is associated with many neurodegenerative diseases [11]. In the initial phase of neurodegeneration, neuroinflammatory responses seem to be protective in order to eliminate pathogens or abnormal proteins. However, with the advanced stages of neurodegenerative changes, the development of chronic inflammation leads to deleterious effects, provoking neuronal dysfunction, synaptic degeneration, and neuronal death [12]. As a confirmation, neuroinflammation has been reported to play a significant role in favoring neurodegeneration in humans, and in vivo studies revealed inflammatory cell activation in patients with AD, other dementias [13,14], and in the MCI condition [15,16]. To underlie dementia-related changes at the earliest possible stage, a large part of recent biomarker research focused on several peripheral inflammatory markers as key elements in neurodegeneration, including multiple cytokines, lymphocytes, neutrophils, other hematological cells and counts, and their related ratios [17]. The ratios are markers of peripheral inflammation that are simply accessible, with high reproducibility and low cost. Thus, they have been largely used in various neurodegenerative conditions [18,19,20]. The most studied ratios are the neutrophil-to-lymphocyte ratio (NLR), the platelet-to-lymphocyte ratio (PLR), the monocyte-to-lymphocyte ratio (MLR), the lymphocyte-to-monocyte ratio (LMR), and the systemic immune inflammation index (SII), defined as platelet count × neutrophil count/lymphocyte count, extensively studied in cancer, stroke, venous sinus thrombosis, and other brain disorders [21,22].

In AD, several studies have reported decreased counts of lymphocytes [23,24]. Consequently, the NLR has been shown to be significantly higher in AD patients than in controls [25,26,27]. However, longitudinal studies reported controversial results [26]. Similarly, PLR has been associated with the development of cognitive impairment [17,23,28], while the results for LMR and MLR are inconclusive [23,28]. In MCI, results are even less conclusive, and the peripheral markers of inflammation are considered not specific enough to be considered a diagnostic marker for AD or MCI [17].

Since widely available and easily accessible screening markers are still lacking in the MCI, this study aimed to explore the role of the NLR and other blood cell counts and related ratios in predicting cognitive decline and conversion to dementia in a large cohort of amnestic MCI participants, followed-up until five years.

## 2. Materials and Methods

### 2.1. Sample Selection

Participants in this retrospective, longitudinal, long-term follow-up study had been referred either to the Centre for Dementia and Cognitive Disorder (CDCD) at the Sant’Andrea Hospital, Vercelli, Italy, or to the Memory Unit, Hospital Clínico Universitario Valladolid, Spain. The inclusion criteria were:

1. amnestic MCI diagnosis according to Petersen criteria [4],
2. age between 65 and 90 years,
3. symptoms’ onset within three years before the first evaluation (baseline),
4. at least one available follow-up visit between two and five years after the baseline evaluation,
5. Mini-mental state examination (MMSE) is available both at baseline and follow-up visits,
6. a blood test performed within three months from the baseline evaluation.

The current study excluded all patients referred to the two Memory Centers with a diagnosis other than amnestic MCI (non-amnestic MCI, dementia, psychiatric disorders, subjective cognitive decline) or lacking a blood test performed within three months from the first evaluation. Other exclusion criteria were:

7. lack of fundamental demographic data (age, educational level) or cognitive assessment at baseline or follow-up,
8. history of active cancer, rheumatic disease, autoimmune disease, chronic infection, hormone therapy, and/or chemotherapy within 15 years from the first evaluation,
9. uncontrolled comorbidities (diabetes, heart failure, respiratory insufficiency, chronic kidney disease),
10. altered C-Reactive Protein (CRP).

**Figure 1:**
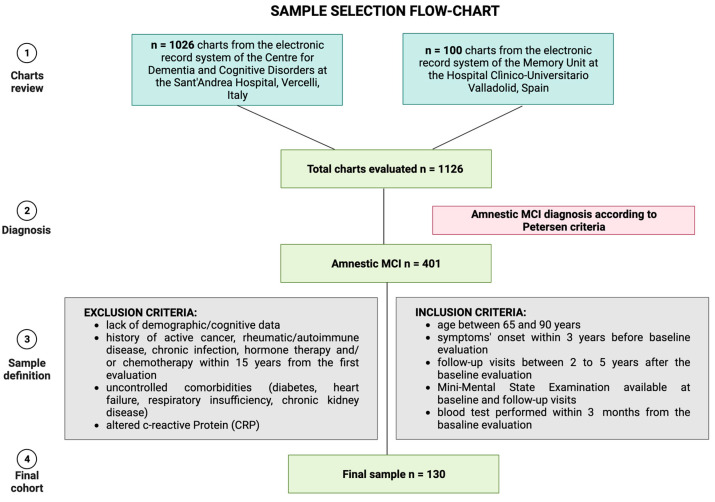
Sample selection strategy. Figure created with Biorender.com.

In detail, the amnestic MCI cohort was obtained by screening *n* = 1126 charts from the electronic health record systems of the two Memory Centers. We included visits between 1 January 2018 to 31 December 2022. We excluded *n* = 725 patients due to other diagnoses than amnestic MCI. By evaluating the clinical data of the remaining *n* = 401 MCI, we included only subjects complying with the inclusion and exclusion criteria, excluding the other *n* = 271 subjects. Figure 1 summarizes the sample selection process.

All available demographic and clinical data were extracted from the selected subjects’ charts and included: age at baseline, sex, educational level, years from symptoms’ onset, comorbidities, actual therapy, and added therapy. Among comorbidities, we considered vascular risk factors and common conditions in the elderly, including anemia, Parkinson’s disease, breathing difficulties, angina, hypertension, diabetes mellitus, peripheral vascular disease, transient ischemic attack, reported history of stroke or heart attack [29], and together with recognized risk factors associated with the development of dementia, including hearing loss, smoking, alcohol consumption, traumatic brain injury, obesity, and depression [30]. In addition, we collected ongoing drugs, focusing on the use of acetylcholinesterase inhibitors as “protective therapies” [31] and benzodiazepines and antidepressants, which are potentially associated with cognitive decline [32,33].

Lastly, we collected the baseline and follow-up primary diagnosis for each included subject, which was evaluated by a neurologist and a psychologist with cognitive impairment and dementia expertise; each diagnosis, including MCI, dementia due to AD, frontotemporal dementia, Lewy bodies dementia, and vascular dementia, was established according to international consensus criteria [4,34,35,36,37].

### 2.2. Hematological Data

Blood sampling was performed in the morning after overnight fasting for all included subjects within three months from the baseline visit. The following hematological tests were considered for the current study: white blood cell (WBC) count (neutrophil, lymphocyte, monocyte, eosinophilia, and basophilia), red blood cell (RBC) count, hemoglobin and hematocrit, platelet count, mean platelet volume, glycemia, blood creatine, total cholesterol, HDL and LDL cholesterol, triglycerides, B12 vitamin, folic acid, thyroid-stimulating hormone (TSH), and CRP. From the blood cell counts, we derived the ratios already tested in MCI populations as follows: the NLR was calculated as the ratio of the neutrophil count to the lymphocyte count; the MLR was calculated as the ratio of the monocyte counts to the lymphocyte count; and PLR was calculated as the ratio of the platelet counts to the lymphocyte count. Lastly, the SII was calculated by the formula: platelet count x neutrophil count/lymphocyte count.

### 2.3. Clinical and Cognitive Baseline-to-Follow-Up Assessment

We used the index of progression as a measure of cognitive decline over time, estimated by the formula: follow-up MMSE—baseline MMSE/years of follow-up. The index of progression represents a reliable measure of cognitive decline over time, allowing us to compare the progression of cognitive deterioration in different groups of MCI subjects [9,38].

Based on the main established diagnosis at the last available follow-up, amnestic MCI subjects were dichotomously classified as CONVERTERS (MCI converting to any type of dementia) and non-CONVERTERS (MCI showing a stable clinical and cognitive status at follow-up). Conversion to dementia was also considered an outcome variable to test the prognostic relevance of baseline clinical and hematologic data.

### 2.4. Statistical Analysis

All continuous data are presented as the mean ± standard deviation (SD). All categorical variables are presented as numbers (percentages). For continuous variables, we used the Kolmogorov–Smirnov test to explore the normality of the distribution of data. Due to the non-normality of the distribution of most variables, the Kruskal–Wallis and the Mann–Whitney U tests were used for continuous data comparison. A chi-square test was performed to compare categorical variables. Statistical significance was set at a *p*-value < 0.05. IBM SPSS software (version 25.0, IBM Corporation, Armonk, NY, USA) was used for the statistical analysis. As a preliminary analysis, the index of progression was used to measure the disease progression rate. Spearman’s correlation was performed to assess the association between hematological data and the index of progression. All clinical, cognitive, and hematological variables were compared between CONVERTERS and non-CONVERTERS. Variables emerging as significantly different between groups, together with variables that emerged as significantly correlating with the index of progression, were considered potential prognostic relevant factors. We then explored the prognostic weight of each relevant variable in a logistic regression analysis, considering conversion as the dichotomous dependent variable and show as the receiver operating characteristic curve. The study was approved by the local Ethic Committee (Ethic Committee Name: Comitato Etico Interaziendale Alessandria; Approval Code: 14415/2023) and performed in compliance with the Declaration of Helsinki.

## 3. Results

### 3.1. Study Sample and Clinical-Hematological Correlations

The final sample included *n* = 130 participants with the MCI diagnosis [4] who met the inclusion criteria. Table 1 summarizes the demographics, clinical, and cognitive characteristics of all participants. The median age of MCI at baseline was 76.50 (72.00–79.00) years, with an educational level of 7.25 ± 3.28 years. N = 72 (55%) were female. The average period of symptoms’ onset was 1.20 (0.60–2.40) years, while subjects were evaluated at a median follow-up of 2.67 (2.33–3.50) years. The baseline MMSE corrected score was 26.02 ± 2.28 points; at follow-up, participants scored on average 23.10 ± 4.32 MMSE points, with a mean of −1.06 ± 1.33 points per year lost.

To explore the relationship between clinical variables and hematological data, we performed Spearman correlation analyses between baseline clinical characteristics and ratios. A weak direct correlation emerged between age at baseline and the NLR (r = 0.29, *p*-value = 0.001); thus, age was considered a nuisance variable in all the subsequent analyses.

### 3.2. Cognitive Decline Progression: Correlation Analysis

**Figure 2:**
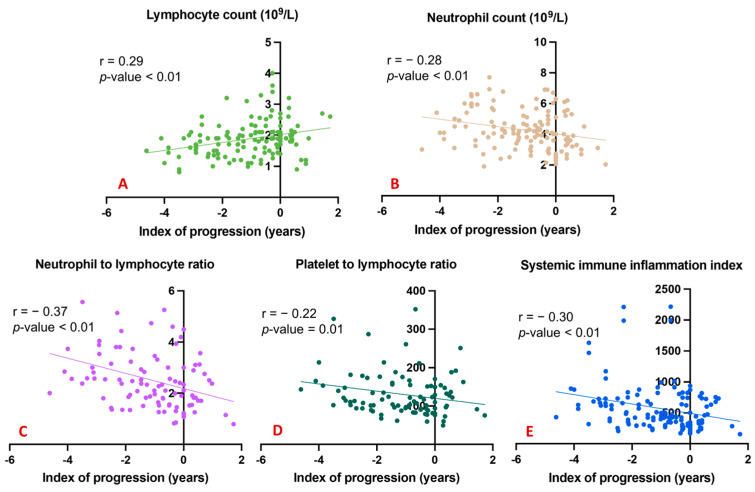
Correlation between index of progression and blood counts and ratios. (**A**) shows a significant direct correlation between the total lymphocyte count and the index of progression; (**B**) shows significant inverse correlations between the index of progression and blood levels of neutrophils; (**C**) shows significant inverse correlations between the index of progression and the NLR; (**D**) shows significant inverse correlations between the index of progression and the PLR; (**E**) shows significant inverse correlations between the index of progression and the SII.

To underline the possible effects of the considered peripheral markers and the degree of cognitive decline over time, we explored correlations between all blood counts and ratios and the index of progression. A significant direct correlation was evident between the total lymphocyte count and the index of progression (Figure 2A); conversely, significant inverse correlations emerged between the index of progression and blood levels of neutrophils (Figure 2B), the NLR (Figure 2C), the PLR (Figure 2D), and the SII (Figure 2E).

### 3.3. Conversion to Dementia: Comparison between Groups

**Figure 3:**
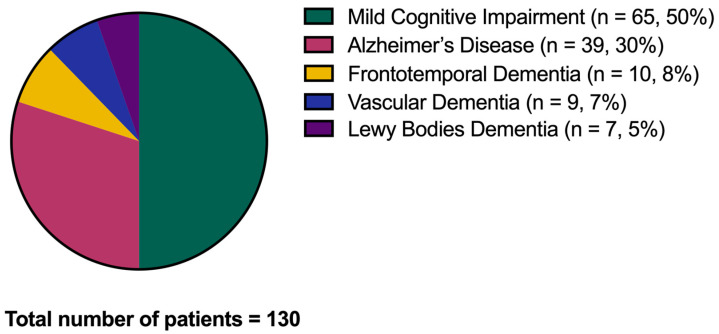
Conversion to dementia. The graph shows the conversion from the Mild Cognitive Impairment condition to dementia over the disease follow-up.

After the considered follow-up, from a minimum of two years to a maximum of five years, *n* = 65 amnestic MCI subjects (50%) converted to dementia (CONVERTERS), and the same portion remained stable in the MCI condition (non-CONVERTERS). Of the 65 CONVERTERS, 39 converted to AD (30% of the whole cohort), 10 to frontotemporal dementia (8%), 9 to vascular dementia (7%), and 7 to Lewy bodies dementia (5%) (Figure 3).

**Figure 4:**
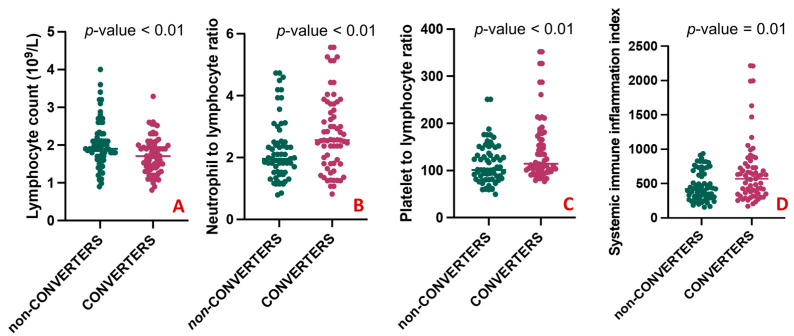
Differences in blood counts and peripheral markers between CONVERTERS and non-CONVERTERS. (**A**) shows a statistically higher number of lymphocytes in CONVERTERS compared with non-CONVERTERS; (**B**–**D**) respectively shows elevated NLR (2.77 ± 1.21 vs. 2.23 ± 0.99; 146.47 ± 69.53 vs. 113.43 ± 42.83 and 673.37 ± 468.88 vs. 471.56 ± 218.04) in CONVERTERS compared to non-CONVERTERS.

A comparison of the clinical characteristics between the two groups is shown in Table 2. There was no statistically significant difference between CONVERTERS and non-CONVERTERS regarding age, sex, educational level, the median time of symptom onset, and the median follow-up. The baseline MMSE mean corrected score was slightly but significantly lower in CONVERTERS than in non-CONVERTERS (25.36 ± 1.97 vs. 26.69 ± 2.37, *p*-value = 0.004). As expected, at the follow-up, in the CONVERTERS group, the MMSE mean corrected score was significantly lower than in the non-CONVERTERS (20.01 ± 3.24 vs. 26.18 ± 2.79, *p*-value < 0.001). The presence of one or more than one comorbid conditions had similar frequencies in the two groups. No differences emerged regarding the use of antidepressants, cholinesterase inhibitors, and anxiolytic therapy. When comparing blood counts and peripheral markers of inflammation among groups, CONVERTERS had a statistically lower number of lymphocytes (1.73 ± 0.49 vs. 2.04 ± 0.62; *p*-value = 0.004) compared with non-CONVERTERS (Figure 4A), but elevated NLR (2.77 ± 1.21 vs. 2.23 ± 0.99; *p*-value = 0.005) (Figure 4B), PLR (146.47 ± 69.53 vs. 113.43 ± 42.83; *p*-value = 0.002) (Figure 4C), and SII levels (673.37 ± 468.88 vs. 471.56 ± 218.04; *p*-value = 0.015) (Figure 4D).

### 3.4. Factors Predicting Conversion to Dementia

**Figure 5:**
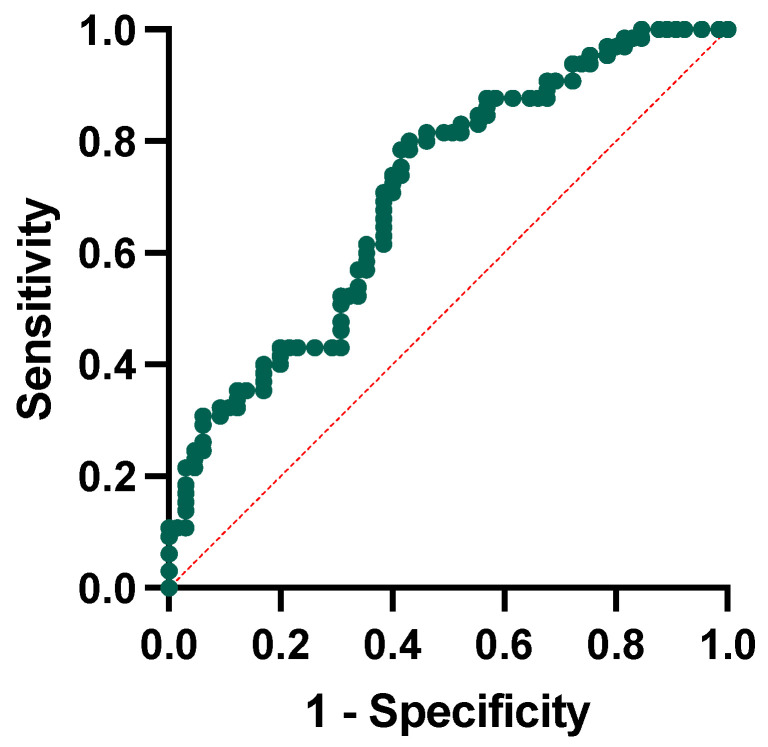
Receiver operating characteristic curve analysis for neutrophil-to-lymphocyte ratio and MMSE corrected score in conversion to dementia.

We conducted a logistic regression analysis to test whether ratios were independent factors associated with conversion to dementia. The regression model included the conversion to dementia as the dependent variable and the following independent variables: the NLR, the PLR, and the SII, the baseline MMSE corrected score, and age at baseline. The model showed that only the NLR (*p*-value = 0.011) and the baseline MMSE score (*p*-value = 0.002) were significantly associated with higher possibilities of conversion to dementia at follow-up. We then performed receiver operating characteristic curve analysis to test the single and combined power of the NLR and MMSE corrected score to predict the conversion to dementia. For the NLR alone, we obtained a cut-off of 2.53 (sensibility of 55% and specificity of 78%), whereas for the MMSE, a corrected cut-off of 25.80 (sensibility of 67% and specificity of 64%). The combination of the included variables has a 65% sensitivity and 62% specificity, with the area under the curve yielding a significant result (AUC = 0.707, *p*-value < 0.001, Figure 5).

### 3.5. Tertiles Analysis

**Figure 6:**
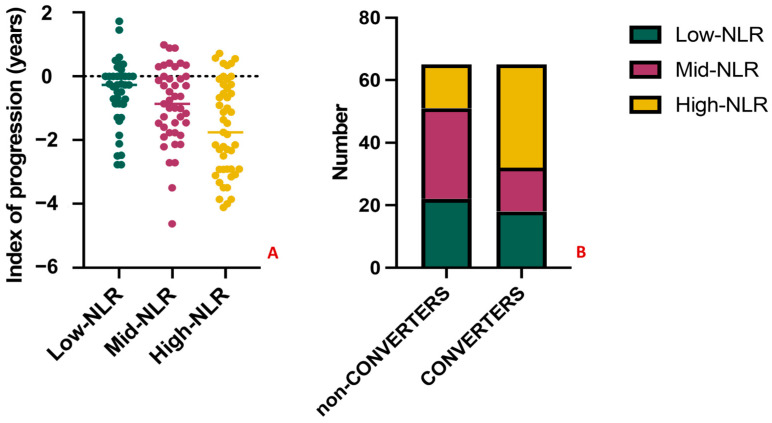
Results of the tertiles analysis. The index of progression and the conversion to dementia significantly differ between amnestic MCI subjects in the High-NLR group (in yellow) when compared with Mid-NLR (purple) and Low-NLR (green) groups. Specifically, subjects in the High-NLR group had the highest loss of MMSE points per year ((**A**), *p*-value = 0.04) and the highest conversion rate (**B**), with *n* = 31 (69%) of MCI converting to dementia at follow-up (*p*-value = 0.005). No significant differences were observed when comparing the Mid-NLR and Low-NLR groups among them (*p*-value > 0.05).

To further explore the predictive significance of the NLR, we performed a tertiles analysis. MCI subjects were divided into three groups according to NLR tertiles in Low-NLR (<1.82), Mid-NLR (1.82–2.56), and High-NLR (>2.56). The variables related with the outcome, namely the index of progression and the conversion to dementia, were compared between groups. Patients in the High-NLR group had significantly worse outcomes (Figure 6A), showing the highest loss of MMSE points per year (−1.54, *p*-value = 0.04) and the highest conversion rate, with *n* = 31 (69%) of MCI converted to dementia at follow-up, vs. *n* = 16 (36%) and *n* = 18 (45%) in the Mid-NLR and Low-NLR, respectively (*p*-value = 0.005) (Figure 6B). Lastly, classifying patients based on the clinical diagnosis, 14/65 (22%) of MCI, 22/39 (56%) of AD, 2/10 (20%) of frontotemporal dementia, 3/9 (33%) of vascular dementia, and 3/7 (43%) of Lewy bodies dementia had NLR in the third tertile (*p*-value = 0.006).

## 4. Discussion

In the present study, we investigated the associations between clinical, cognitive, and laboratory data with the progression of cognitive decline and the conversion to dementia in a large group of amnestic MCI subjects. Our results suggested that a higher NLR in peripheral blood at the time of MCI diagnosis was associated with a faster cognitive decline and a higher conversion rate to dementia. Furthermore, considering the reported association between neuroinflammation and neurodegeneration in AD and MCI conditions, we hypothesized that the NLR, and especially extremes of the NLR, could have prognostic value in identifying amnestic MCI at risk for conversion.

Dysregulation of the neuroinflammatory responses has been well documented in AD and MCI. Neutrophils have been suggested as essential players associated with AD pathogenesis, showing increasing numbers and activation [39]. A reduced lymphocyte count has been reported in AD, and, contextually, older adults with AD have a higher NLR than healthy controls [24,25,40]. Changes in leukocyte counts may be related to AD development in several pathways; high levels of neutrophils are hallmarks of chronic inflammation. In contrast, low lymphocyte levels may indicate a reduced ability to respond to pathogens, both representing potential markers of early dementia stages [40]. Another possible involved mechanism could be related to an immunosenescent process directly caused and sustained by AD pathology through a change in the autonomic system and an alteration of the neuroendocrine response. This hypothesis is supported by previous findings, mainly in ischemic stroke. It has been demonstrated that an immunosuppression condition after the acute vascular event favors the inflammatory catecholamines released into the blood through an over-activated sympathetic system, which may alter the peripheral blood population, reducing circulating lymphocytes [41]. The NLR and other ratios represent composite inflammatory markers providing information from two or more leukocyte populations, thus integrating multiple possible dysregulation pathways. In addition, the measurement of cell counts and the ratio is widely available in all hospitals, representing a routinary tool in the baseline assessment of individuals with cognitive decline. The accessibility and reproducibility of biomarkers represent the most critical challenge for the future development of therapeutic strategies for dementia, especially in an early phase [42].

In the current study, we investigated the prognostic role of peripheral inflammatory markers in MCI, showing correlations between the annual changes in the MMSE—expressed as points lost per year—and several blood counts and ratios, including neutrophils, lymphocytes, the NLR, the PLR, and the SII. To our knowledge, this is the first evidence in a large amnestic MCI population to explain a relationship between cognitive decline over time and dysregulation in neutrophil and lymphocyte counts and their related ratios. Similar evidence has been obtained in a recent study showing that the NLR correlated with cognitive impairment in individuals with cerebral small vessel disease [43]. In a second step, to identify variables associated with the conversion to dementia, we classified MCI subjects according to the primary follow-up diagnosis in stable MCI (non-CONVERTERS) and demented patients (CONVERTERS). Several baseline differences emerged between the two groups regarding hematological data: a lower number of lymphocytes in the CONVERTERS group, besides higher NLR, PLR, and SII. These results again confirm the presence of a dysregulated peripheral inflammatory mechanism in amnestic MCI, which will convert to dementia, sustained by a lower number of lymphocytes and an increased level of inflammatory indexes. The only clinical-cognitive parameter showing differences between CONVERTERS and non-CONVERTERS was the baseline MMSE score, which was slightly lower in people converting to dementia at the follow-up. This is an expected result since several studies reported the MMSE as a tool for the prediction of developing dementia in people with MCI; however, given the evidence from the literature, the MMSE cannot be used as a stand-alone single-administration test to predict conversion to dementia in the future [44].

To test the independent factors associated with conversion to dementia, we operated a logistic regression with several independent variables (NLR, PLR, SII, baseline MMSE corrected score, and age at baseline), and only the NLR and the baseline MMSE score were significantly associated with a higher risk of conversion to dementia at follow-up. The prognostic role of the NLR was confirmed in the subsequent tertile analysis, showing that MCI with a higher NLR had significantly worse outcomes, either as the highest conversion rate or faster cognitive decline over time. Interestingly, the negative outcome is observed only in the High-NLR group, while no differences in terms of conversion risk are observed between the Low-NLR and the Mid-NLR tertiles. Models using tertiles have been proposed to stratify the values of various indexes, including the NLR, in different neurodegenerative conditions to test the extremes’ role in predicting outcomes [19,20].

The receiver operating characteristic curve analysis testing the power of the NLR and MMSE corrected score at baseline in predicting conversion yielded significant results with moderate sensitivity and fair specificity (AUC = 0.707). This result is in line with those reported in the literature. Previous studies evaluating the NLR in MCI populations reported contrasting results indeed. The first evidence emerged from the work of Rembach and colleagues, evaluating the NLR in AD, MCI, and controls [26]. The NLR was evaluated longitudinally, showing a weak correlation with the amyloid burden. In addition, the cross-sectional evaluation of the NLR showed higher values in AD than in controls; in contrast, the longitudinal evaluation between people converting to dementia showed no significant differences, suggesting a suboptimal performance of the NLR for diagnostic and prognostic purposes [26]. The uncertain prognostic role of the NLR has been suggested by other studies, reporting significantly lower NLR values in AD patients and MCI when compared with controls but without showing differences to distinguish AD from MCI [23,27]. More recently, An and colleagues explored the role of the NLR in MCI subjects, enrolling a population similar to that of our study (*n* = 186). The NLR in MCI was significantly higher than in controls, and the elevated NLR was significantly associated with an increased risk of MCI [45]. Lastly, studies exploring correlations between peripheral markers and cognitive, neuropsychological, and neuroimaging data in large datasets showed that NLR values directly correlate with cognitive decline; furthermore, higher values of NLR represented a risk factor for subsequent dementia, together with age and cerebrovascular risk factors [40,46].

Our study has some limitations, and the main one regards the lack of biomarkers to confirm baseline and follow-up diagnoses and stratify subjects. We solely considered the clinical diagnosis and classified subjects at follow-up as patients with or without dementia. However, MCI is a heterogenous condition that can be associated with multiple etiologies. We used stringent inclusion criteria to overcome this limitation and corrected the analyses for age and sex. To reduce the heterogeneity of the sample, we included only subjects with amnestic MCI, which is the most common onset of AD. To limit the effect of age as a confounding factor, we included only subjects over 65 years, given the possible influence of aging on blood cell counts [47], and corrected the main analyses for age. In addition, since conversion to dementia in MCI populations is associated with follow-up and disease duration, we included a homogenous follow-up comprised between two and five years [48,49]. Since comorbidities may influence cognitive decline [50,51,52], we analyzed differences for several comorbidity rates and blood parameters, resulting in no differences between groups. Lastly, we are aware that NLR and other ratios can be affected by several confounding factors; still, the peripheral markers of inflammation may reflect an imbalanced inflammatory status.

## 5. Conclusions

Our results suggest that peripheral inflammatory markers and immune profiles can be an easily accessible tool to stratify MCI subjects according to their risk of developing dementia. In particular, higher values of NLR are associated with a steeper cognitive decline over time and a higher risk of conversion to dementia within five years of diagnosis. Integrating the information related to the blood cell counts provided with clinical and cognitive measures, including the MMSE at baseline, may represent an efficient strategy to intercept subjects at risk for conversion early in the disease course.

## Figures and Tables

**Table 1:** Subject demographic, clinical, and cognitive characteristics.

Sample Characteristics	Amnestic MCI (*n* = 130)
Age at the first evaluation (median ± IQR)	76.50 (72.00–79.00) years
Female sex, *n* (%)	72 (55%)
Educational level (mean ± SD)	7.25 ± 3.28 years
Symptom’s onset (median ± IQR)	1.20 (0.60–2.40) years
Follow-up duration (median ± IQR)	2.67 (2.33–3.50) years
Baseline MMSE corrected score (mean ± SD)	26.02 ± 2.28 points
Follow-up MMSE corrected score (mean ± SD)	23.10 ± 4.32
Index of progression	−1.06 ± 1.33 points per year
Conversion at the last follow-up, *n* (%)	65 (50%)

Characteristics of the whole amnestic MCI sample. Symptom’s onset represents the timeframe (in years) between the reported symptom’s onset and the first clinical evaluation. Index of progression is calculated by the formula: (follow-up MMSE score − baseline MMSE score)/years of follow-up. Abbreviation: Mild Cognitive Impairment: MCI; mini-mental state examination: MMSE; IQR: interquartile range; Standard Deviation: SD.

**Table 2:** Comparison among demographic, clinical, cognitive, hematological, and biochemistry characteristics between non-CONVERTERS and CONVERTERS.

Demographics and Clinical Data	Non-Converters *n* = 65	Converters *n* = 65	*p*-Value
Age at the first evaluation (mean ± SD)	75.42 ± 5.08 years	76.83 ± 5.07 years	0.169
Female sex, *n* (%)	31 (48%)	41 (63%)	0.077
Educational level (mean ± SD)	7.02 ± 3.07 years	7.49 ± 3.47 years	0.342
Symptom’s onset (mean ± SD)	1.15 ± 0.83 years	1.10 ± 0.76 years	0.783
Follow-up duration (mean ± SD)	2.84 ± 0.87 years	3.01 ± 0.73 years	0.119
Subjects with no or one comorbidity, *n* (%)	21 (32%)	21 (32%)	-
Subjects with two or more comorbidities, *n* (%)	44 (68%)	44 (68%)	-
**Cognitive data**			***p*** **-value**
Baseline MMSE corrected score (mean ± SD)	26.69 ± 2.37 points	25.36 ± 1.97 points	**0.004**
Follow-up MMSE corrected score (mean ± SD)	26.18 ± 2.80 points	20.01 ± 3.24 points	**<0.001**
Index of progression	−0.19 ± 0.73 points per year	−1.93 ± 1.23 points per year	**<0.001**
**Hematological and biochemistry data**			***p*** **-value**
WBCs (10^9^/L)	6.69 ± 1.60	6.78 ± 1.42	0.495
Neutrophils (10^9^/L)	4.17 ± 1.26	4.89 ± 1.35	0.328
Lymphocytes (10^9^/L)	2.94 ± 0.62	1.73 ± 0.49	**0.001**
Monocyte (10^9^/L)	0.45 ± 0.12	0.40 ± 0.12	0.054
Eosinophilia (10^9^/L)	0.18 ± 0.11	0.17 ± 0.10	0.406
Basophilia (10^9^/L)	0.04 ± 0.04	0.03 ± 0.04	0.533
RBCs (10^12^/L)	4.34 ± 0.50	4.30 ± 0.48	0.192
Hemoglobin (g/dL)	13.64 ± 1.33	13.55 ± 1.47	0.849
Hematocrit (%)	42%	41%	0.732
Platelet (10^9^/L)	213.52 ± 50.81	231.64 ± 64.92	0.167
Mean platelet volume (femtoliters)	8.76 ± 1.70	8.57 ± 1.63	0.418
NLR	2.23 ± 0.99	2.77 ± 1.21	**0.005**
LMR	4.81 ± 1.73	4.82 ± 2.84	0.416
MLR	0.23 ± 0.08	0.24 ± 0.008	0.416
PLR	113.43 ± 42.83	146.47 ± 69.53	**0.002**
SII	471.56 ± 218.04	673.37 ± 468.88	**0.015**
Fasting glucose (mg/dL)	100.45 ± 25.98	99.18 ± 31.15	0.614
Creatinine (mg/dL)	0.88 ± 0.15	0.85 ± 0.21	0.721
Total Cholesterol (mg/dL)	172.30 ± 56.79	180.49 ± 53.25	0.393
Triglycerides (mg/dL)	120.97 ± 60.81	115.37 ± 47.65	0.899
Vitamin B12 (pg/mL)	380.95 ± 258.10	345.73 ± 251.20	0.335
Folate (ng/mL)	6.58 ± 4.53	6.28 ± 4.54	0.829
Thyroid-stimulating hormone (mIU/L)	1.84 ± 1.76	1.31 ± 0.99	0.109
C-Reactive Protein (mg/dL)	0.34 ± 0.10	0.32 ± 0.12	0.784

All continuous data are presented as the mean ± standard deviation (SD). All categorical variables are presented as numbers (percentages). For continuous variables, we used the Mann–Whitney U test; the chi-square test was performed to compare categorical variables. Statistical significance was set at a *p*-value < 0.05. Statistically significant results are reported in bold. Abbreviations: lymphocyte-to-monocyte ratio: LMR; mini-mental state examination: MMSE; monocyte-to-lymphocyte ratio: MLR; neutrophil-to-lymphocyte ratio: NLR; platelet-to-lymphocyte ratio: PLR; red blood cells: RBCs; systemic immune inflammation index: SII; Standard Deviation: SD; white blood cells: WBCs.

## Data Availability

Data will be made available on request.
